# The American Medical Association—The Code

**Published:** 1883-07-20

**Authors:** 


### *THE AMERICAN MEDICAL ASSOCIATION—THE CODE.*

At the American Medical Association a paper was read from the St. Louis Medical Society asking for the appointment of a committee to take into consideration a revision of the Code and report at the session of 1884—the committee to be authorized to submit a New Code. The resolution was promptly laid on the table by a decided majority.

The officers of the American Medical Association for the present year are as follows: Austin Flint, Sr., President. R. A. Kinlock, of South Carolina, 1st Vice-President; T. B. Lester, of Missouri, 2d Vice-President; A. L. Gihon, U. S. N., 3d Vice-President; S. C. Gordon, of Maine, 4th Vice-President; D. W. Prentis, of Washington, Secretary; R. J. Dungleson, Philadelphia, Treasurer; C. H. Kleinschmidt, Washington, D. C., Librarian.

The next meeting of the Association will be in Washington, D. C., the first Tuesday in May, 1884.

